# Supplementary material for: Metabolic effects of bezafibrate in mitochondrial disease
Source: EMBO Mol Med. 2020 Feb 28;12(3):e11589. doi: 10.15252/emmm.201911589 (PMC7059007; doi:10.15252/emmm.201911589)
Supplement: Supplementary file 1 — Appendix [file EMMM-12-e11589-s001.pdf]

Appendix

Table of Contents

Appendix Tables S1- S6

Appendix Figures S1 – S4

Appendix Tables

Appendix Table S1. Study entry criteria

The participant is willing and able to give informed consent for participation in the trial.
Male or Female, aged 18 years or over and less than 65 years at the time of screening.
Confirmed mt.3243A>G mutation.
Evidence of myopathy as determined by the investigator.
Stable dose of current regular medication for at least 4 weeks prior to trial entry.
Not already taking fibrates.
No history of liver impairment.
Normal renal function, with a creatinine clearance of >60ml/minute.
Female participants of childbearing potential and male participants whose partner is of childbearing potential must be willing to ensure that they, and/or their partner, use effective contraception during the trial and for 3 months thereafter. In female participants, this may not include oestrogen containing contraceptives.
Willing to allow his or her General Practitioner and consultant, if appropriate, to be notified of participation in the trial.
In the Investigator's opinion, is able and willing to comply with all trial requirements.
Participants who have participated in another research trial involving an investigational product in the past 12 weeks.

Appendix Table S2. Study exclusion criteria

Unstable or poorly controlled diabetes, as determined by the investigator. Participants assigned to group 2 dosing with diabetes (insulin or non-insulin dependent) or glucose intolerance who are unwilling or unable to monitor blood glucose levels during the 12 week treatment period.
History of rhabdomyolysis.
History of sensitivity to fibrates (including photosensitivity).
Hypoalbuminaemia.
Gallbladder disease with or without cholelithiasis.
History of liver impairment or disease.
Prolonged alcohol misuse as determined by the investigator.
Nephrotic syndrome.
Significant renal impairment, or creatine clearance <60ml/minute.
Untreated hypothyroidism.
Use of another medication that interacts with bezafibrate:<br>colestyramine, anion exchange resins, coumarin anticoagulants, monoamine oxidase inhibitors, oestrogens, immunosuppressants, statins.
For MRI – any contra-indication to MRI scanning.
For exercise testing – any participant with ischaemic heart disease.
A female participant who is pregnant, lactating or planning pregnancy during the course of the trial or a male participant who is planning to conceive with their female partner.
Elective or emergency surgery in the past 12 weeks.
Scheduled elective surgery or other procedures requiring general anaesthesia during the trial.
Any other significant disease or disorder which, in the opinion of the Investigator, may either put the participants at risk because of participation in the trial, or may influence the result of the trial, or the participant’s ability to participate in the trial.

Appendix Table S3. Calculation of the human equivalent dose of bezafibrate based on animal studies

Subject	A<br>Human weight (kg)	B<br>Licensed BZF Dosing (mg/kg/day)	C<br>Body Surface Area (m^2)	D<br>Human Km factor	E<br>Mouse Km factor	F<br>HED[1] (mg/kg/day)	G<br>HED[2] (mg/kg/day)
P1	40	15	1.6	25	3	36	60
P2	45	13.3	1.6	28	3	32	54
P3	50	12	1.6	31	3	29	48
P4	60	10	1.6	37	3	24	40.5
P5	70	8.6	1.6	44	3	20	34
P6	80	7.5	1.6	50	3	18	30

[1] - animal diet enriched with bezafibrate at 300mg/kg/day
[2] - animal diet enriched with bezafibrate at 500mg/kg/day

Column A – theoretical participant weights.
Column B - licensed bezafibrate dosing (200mg TDS) expressed in mg/kg/day for participant weights.
Column C - approximate human body surface area.
Column D - calculated human Km factor, where Km is a correction factor (weight (kg)/BSA(m^2)).
Column E - mouse Km factor (Food and Drug Administration, 2005).
Column F – calculated HED of bezafibrate in mg/kg/day using 300mg/kg/day animal dose.
Column G - calculated HED of bezafibrate in mg/kg/day using 500mg/kg/day animal dose.

Appendix Table S4. Body mass index (BMI) of the participants before and after treatment with bezafibrate

The mean BMI did not change during the study.

Subject	Height (m)	Weight (kg)			BMI		
		W0	W6	W12	W0	W6	W12
**P1**	1.56	70.60	70.10	70.20	29	29	29
**P2**	1.66	52.40	53.70	53.75	19	19	20
**P3**	1.73	72.60	73.40	73.10	24	25	24
**P4**	1.76	55.75	55.50	57.90	18	18	19
**P5**	1.67	95.70	95.60	97.90	34	34	35
**P6**	1.63	50.05	49.30	50.20	19	19	19

Appendix Table S5. Non-fasting plasma triglyceride levels (mmol/L, normal <1.7 mmol/L)

P1-P6 = the six participants before and after 12 weeks of treatment. The samples were taken at the same time each day. However, the study protocol did not allow a fasting sample to be taken. There was a trend for bezafibrate to reduce the triglyceride levels, but this did not reach statistical significance (*P*-value = 0.08).

Subject	W0	W12
P1	2.2	1.3
P2	0.8	0.6
P3	1.5	1.0
P4	1.4	1.1
P5	3.8	1.8
P6	1.5	1.5
**Mean**	**1.9**	**1.2**

Appendix Table S6. Quadruple immunofluorescence fibres quantification

Subject	Number of Fibres		Complex I Down (%)		Complex I Negative		Complex IV Down (%)		Complex IV Negative (%)	
	W0	W12	W0	W12	W0	W12	W0	W12	W0	W12
P1	916	716	22.27	10.34	10.81	6.70	7.10	3.49	1.42	0.14
P2	746	1,377	2.54	1.31	2.14	0.07	2.54	0.87	0.13	0.07
P3	598	1,377	24.25	16.07	9.53	4.83	2.17	1.01	0.67	0.22
P4	538	710	21.75	5.92	6.32	2.96	1.30	0.70	0.37	0.14
P5	1,497	334	10.55	16.85	6.88	9.88	1.54	1.74	0.40	0.00
P6	119	1,013	79.82	70.88	57.14	48.57	20.17	9.58	3.36	2.57

Appendix Table S7. Newcastle Mitochondrial Disease Score for Adults (NMDAS) before and after treatment

Each section contains a number of sub-sections which are scored from 0 to 5, with 5 being the most severe. Section 1 includes 10 sub-sections (maximum score 50), Section 2 includes 9 sub-sections (maximum score – 45), Section 3 includes 10 sub-sections (maximum score – 50), giving an overall maximum score of 145 in the most severely affected patients. The sub-scores for the muscle-specific sections are shown in the lower table. P1-P6 = the six participants before and after 6 and 12 weeks of treatment with bezafibrate. There were no significant changes in the mean score in each section, nor the specific scores for exercise tolerance and myopathy.

**Subject**	**Total NMDAS**			**Section 1**			**Section 2**			**Section 3**		
	**W0**	**W6**	**W12**	**W0**	**W6**	**W12**	**W0**	**W6**	**W12**	**W0**	**W6**	**W12**
P1	36	34	31	19	16	13	15	16	15	2	2	3
P2	18	14	11	7	3	2	5	6	4	6	5	5
P3	33	32	31	12	11	9	15	15	16	6	6	6
P4	19	22	16	7	10	4	9	9	11	3	3	1
P5	21	20	22	4	3	4	9	9	10	8	8	8
P6	19	21	17	7	10	7	5	4	4	7	7	6
**Mean**	**24**	**24**	**21**	**9**	**9**	**7**	**10**	**10**	**10**	**5**	**5**	**5**

Subject	Muscle Specific NMDAS					
	Section 1: Exercise Tolerance			Section 3: Myopathy		
	W0	W6	W12	W0	W6	W12
**P1**	3	2	2	1	1	1
**P2**	2	1	1	3	3	3
**P3**	1	2	1	1	1	1
**P4**	3	3	3	3	3	1
**P5**	1	1	1	3	3	3
**P6**	1	1	1	3	3	2
**Mean**	**1.8**	**1.7**	**1.5**	**2.3**	**2.3**	**1.8**

Appendix Figure S1

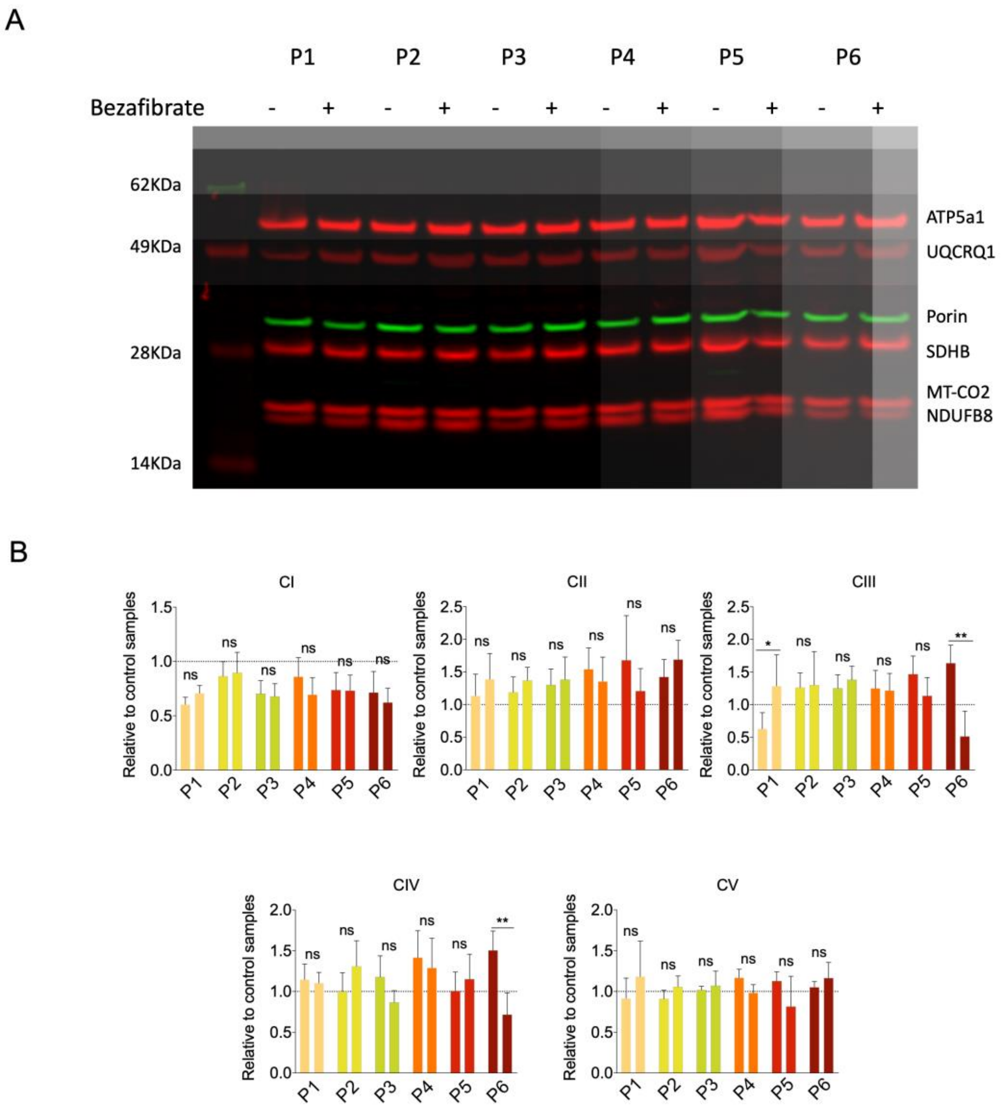

Appendix Figure S1. Mitochondrial enzyme complex quantification in individual patients before and after treatment. Mitochondrial OXPHOS complex protein levels in individual patients before and after bezafibrate treatment. (**A**) Representative image of an immunoblot. Individual patients before (-) and after 12 weeks (+) of treatment for CI = complex I (NDUFB8); CII = complex II (SDHB); CIII = complex III (UQCRC2); CIV = complex IV (COX-II); CV = complex V (ATP5A). Porin used as a loading control to enable quantification independent from the number of mitochondria. (**B**) Semi-quantitative, densitometric analysis of mitochondrial proteins by western blotting normalized to β-Actin levels in individual patients before (plain bars) and after 12 weeks (dotted bars) of treatment. Horizontal dotted lines denote the mean values in healthy age-matched controls. Bars and error bars represent the mean ± SD of the technical replicates at each time point. Statistical testing was performed by using the Mann-Whitney test with an empirical level of significance (****P*-value ≤ 0.001, ***P*-value ≤ 0.01, **P*-value ≤ 0.05).

Appendix Figure S2

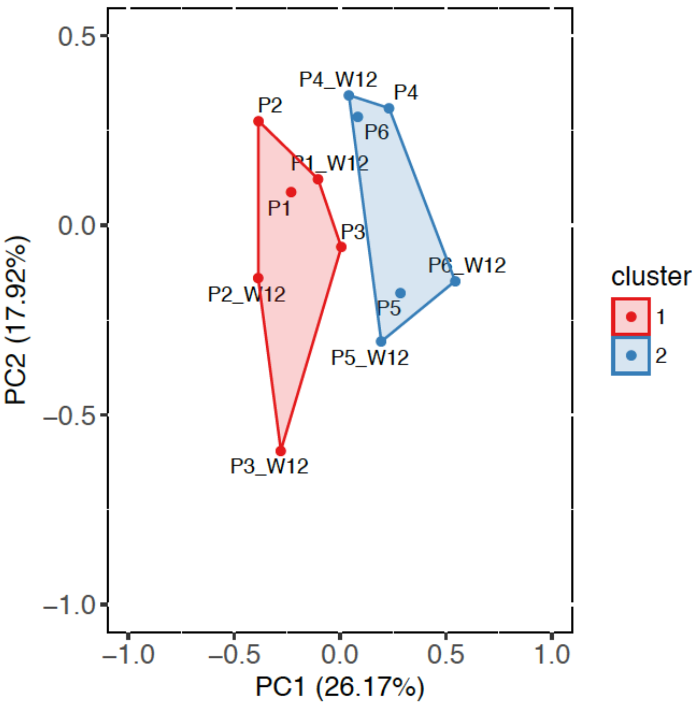

Appendix Figure S2. Transcriptomic response to Bezafibrate. Unsupervised k-means cluster analysis of muscle RNA-seq data identified two groups, but no differences between treated and untreated patients were detected.

Appendix Figure S3

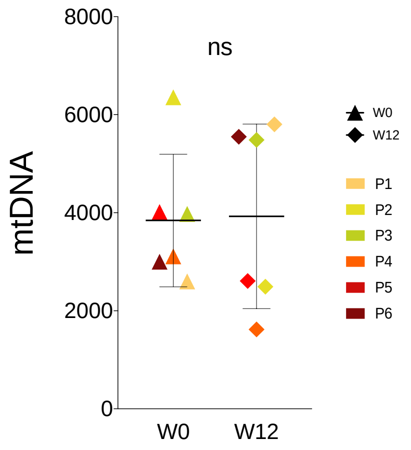

Appendix Figure S3. Mitochondrial DNA copy number. Data recorded in individual patients before and after 12 weeks of treatment. Colour codes represent different samples. Solid horizontal lines and error bars denote the mean ± SD of the samples at different time points. No significant differences were detected before and after treatment using the Wilcoxon signed-rank test with an empirical level of significance.

Appendix Figure S4

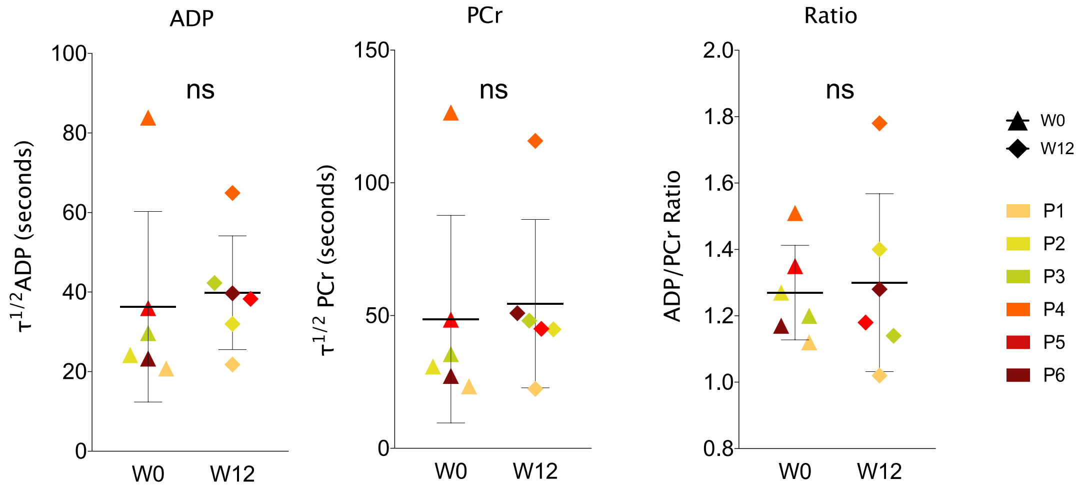

Appendix Figure S4. Skeletal muscle ^{31}P-MRS. Data recorded in individual patients before and after 12 weeks of treatment. Colour codes represent different samples. Solid horizontal lines and error bars represent the mean ± SD of the samples at different time points. No significant differences were detected before and after treatment using the Wilcoxon signed-rank test with an empirical level of significance.
