# Supplementary material for: Metabolic effects of bezafibrate in mitochondrial disease
Source: EMBO Mol Med. 2020 Feb 28;12(3):e11589. doi: 10.15252/emmm.201911589 (PMC7059007; doi:10.15252/emmm.201911589)
Supplement: Supplementary file 2 — Expanded View Figures PDF [file EMMM-12-e11589-s002.pdf]

Expanded View Figures

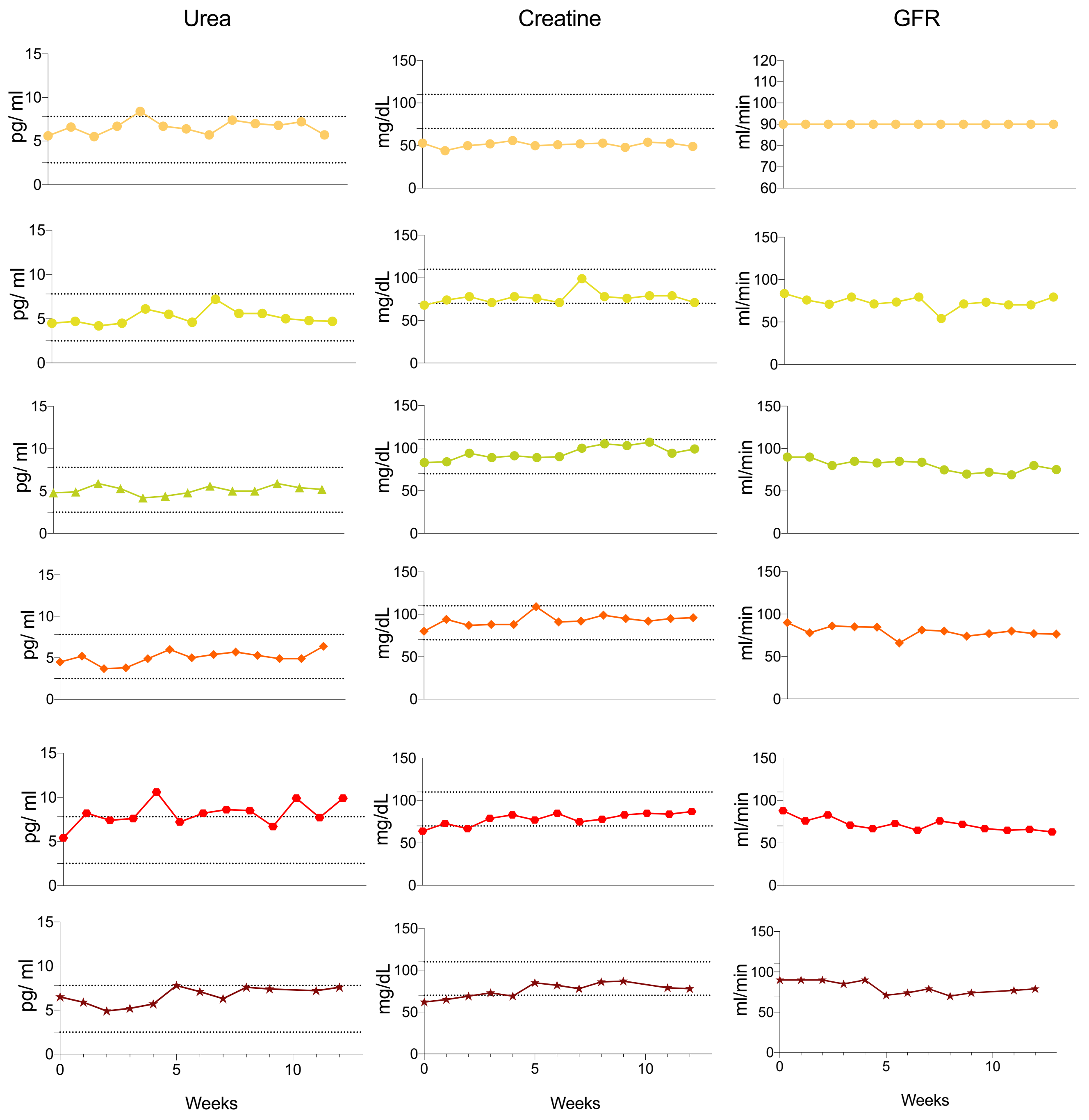

Figure EV1. Additional clinical characteristics of the six patients with the m.3243A>G *MTTL1*.

Renal function of the participants: urea, creatine and estimated glomerular filtration rate (GFR). Dotted lines represent the mean ± SD.

© 2020 The Authors

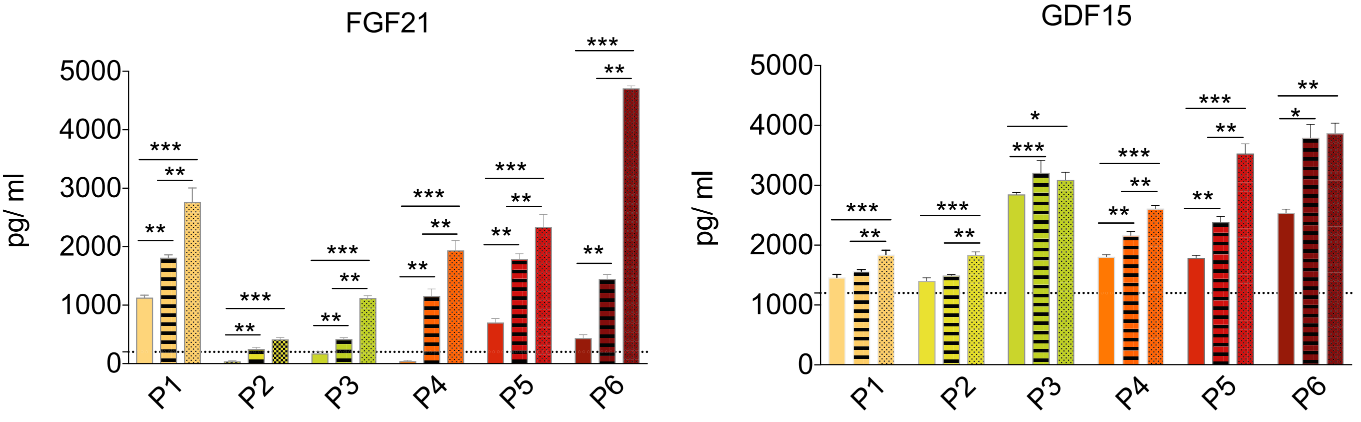

Figure EV2. Serum FGF-21 and GDF-15 levels before, during and after treatment.

Colour code and patterns represent different samples at different time points. Plain bars specify patients before treatment, whereas striped and dotted bars represent the same patients at 6 and 12 weeks after treatment, respectively. Horizontal dotted lines denote the mean values in healthy age-matched controls. Bars and error bars represent the mean ± SD of the technical replicates at each time point. Statistical testing was performed by using the Kruskal–Wallis test followed by Tukey honest significant differences procedure on the ranked data (Serum FGF-21, 6 vs. 0 weeks, P1–P6: *P*-value = 0.0045; 12 vs. 6 weeks, P1, P3, P4, P5 and P6: *P*-value = 0.0045; 12 vs. 0 weeks, P1–P6: *P*-value = 3×10^{-5}; GDF-15, 6 vs. 0 weeks, P3–P6: *P*-value = 0.0067, 0.0045 and 0.0177; 12 vs. 6 weeks, P1, P2, P4 and P5: *P*-value = 0.0041, 0.0045, 0.0045 and 0.0045; 12 vs. 0 weeks, P1–P6, *P*-value: 2×10^{-5}, 3×10^{-5}, 0.0172, 3×10^{-5}, 3×10^{-5}, 0.0053) (****P*-value ≤ 0.001, ***P*-value ≤ 0.01, **P*-value ≤ 0.05).

© 2020 The Authors

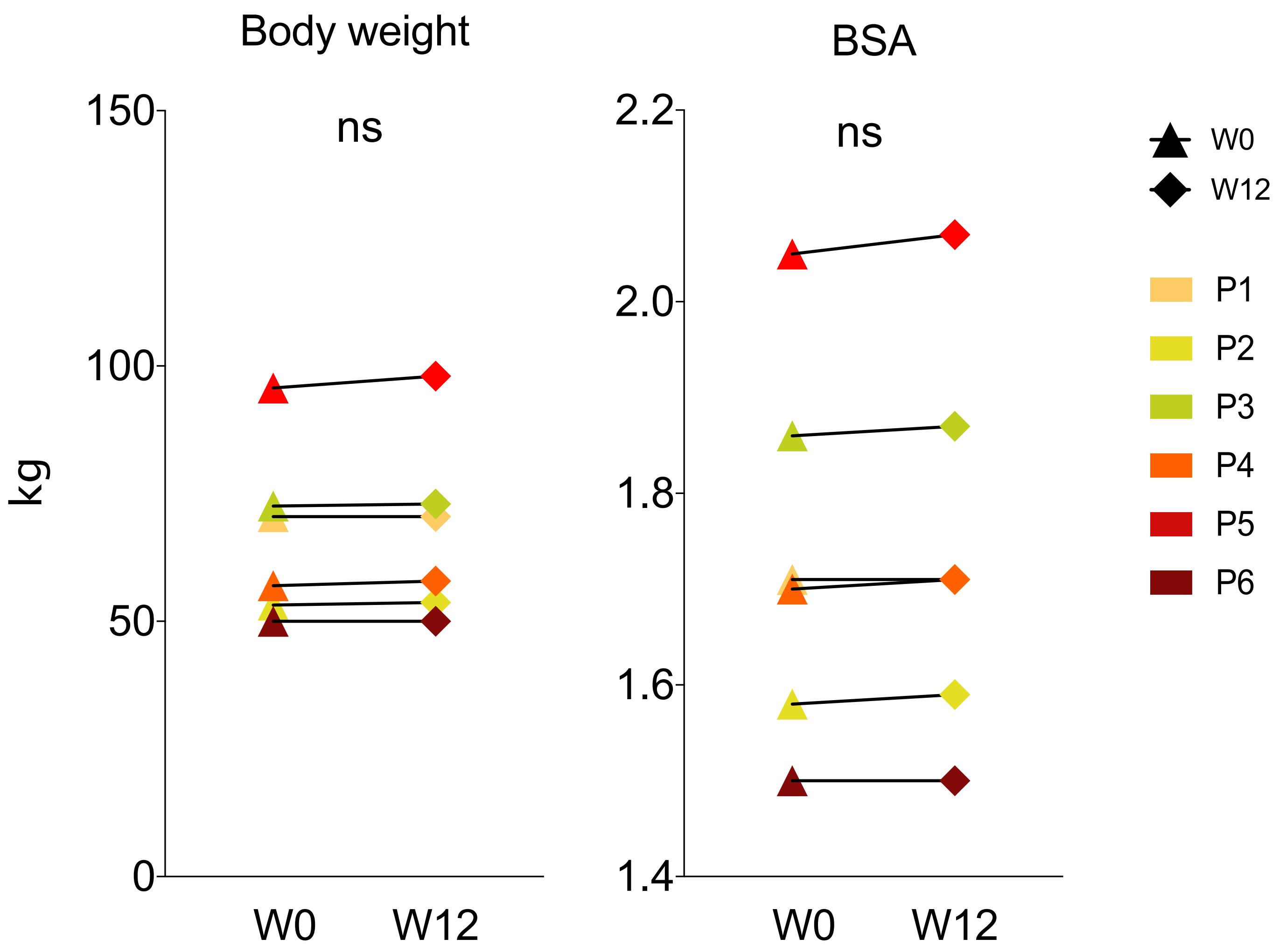

Figure EV3. Additional clinical characteristics of the six patients with the m.3243A>G *MTTL1*.

Bodyweight and basal surface area (BSA) before and after 12 weeks of treatment. Colour codes represent different samples. No significant differences were detected before and after treatment using the two-sided Wilcoxon signed-rank test with an empirical level of significance.

© 2020 The Authors

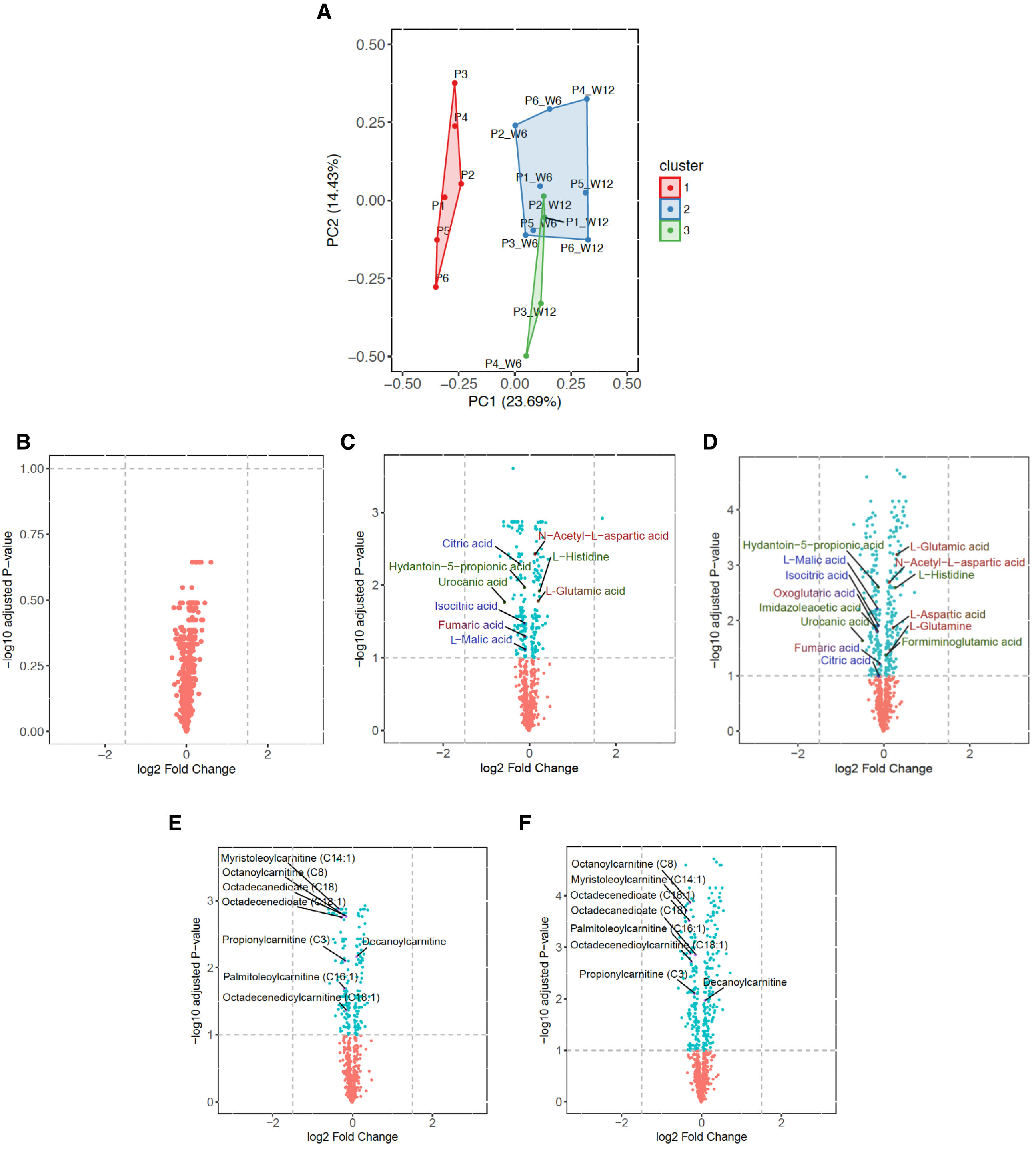

Figure EV4. Metabolic response to Bezafibrate.

A Unsupervised *k*-means cluster analysis of serum metabolites identified three groups: untreated (group 1 in red) and two groups for the treated patients (groups 2 and 3 in blue and green, respectively).

B–D Volcano plot of the differentially regulated metabolites (FDR < 0.1) with (B) no differences observed between 6 weeks and 12 weeks of treatment, (C) after 6 weeks and (D) after 12 weeks. Metabolites in “Alanine, aspartate and glutamate metabolism”, “Histidine metabolism” and “Citrate cycle (TCA cycle)” are highlighted in dark red, dark green and dark blues, respectively, with transition colour code if the metabolite belongs to more than one KEGG pathways.

E, F Volcano plot with differentially regulated metabolites (FDR < 0.1) of the acyl-carnitine metabolites (E) after 6 weeks and (F) 12 weeks.

 © 2020 The Authors

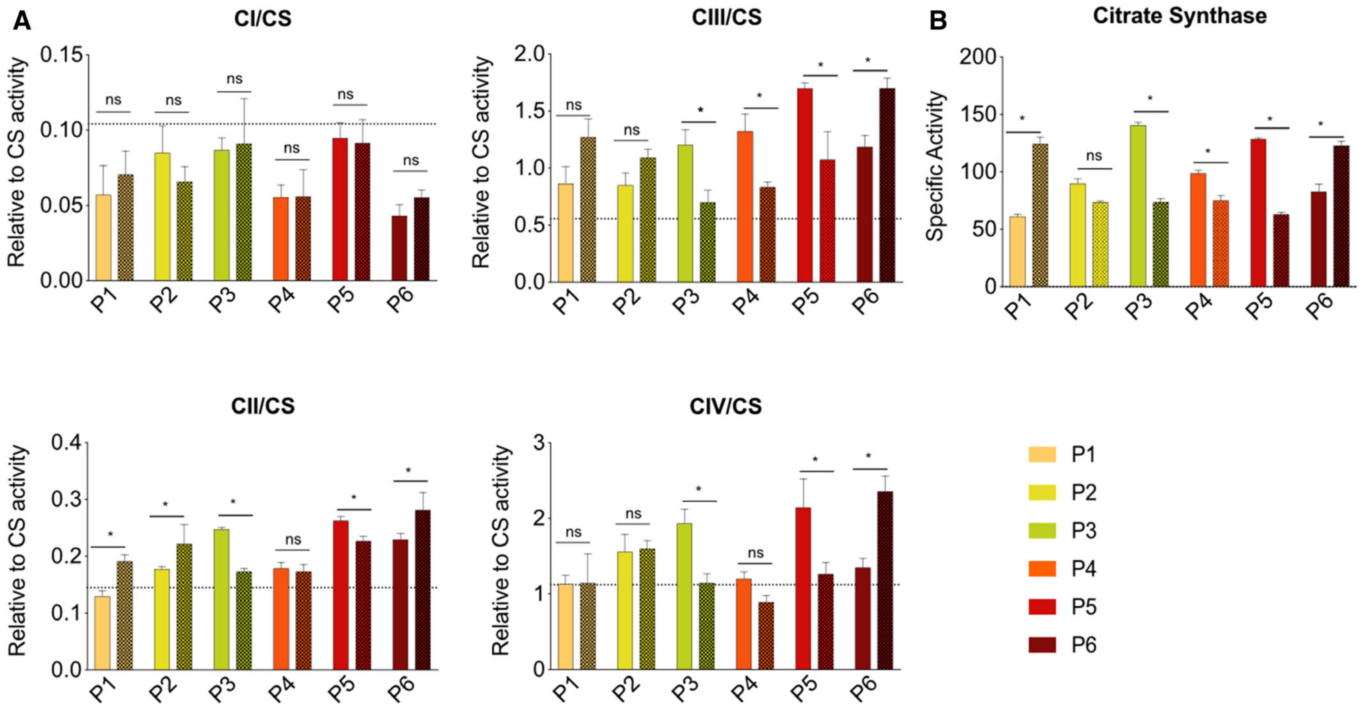

Figure EV5. Mitochondrial enzyme activity in individual patients before and after bezafibrate treatment.

A, B (A) Mitochondrial respiratory chain enzyme activities (expressed relative to citrate synthase activity) and (B) citrate synthase activity (expressed relative to total protein) in individual patients before (plain bars) and after 12 weeks (dotted bars) of treatment. Horizontal dotted lines indicate the mean values in healthy age-matched controls. Bars and error bars represent the mean ± SD of the technical replicates at each time point. Statistical testing was performed by using the two-sided Mann–Whitney test with an empirical level of significance (CII/CS, P1, P2, P3, P5, P6, empirical *P*-value = 0.0318, 0.0360, 0.0260, 0.0354 and 0.0018; CIII/CS, P3–P6, empirical *P*-value = 0.0376, 0.0360, 0.0324 and 0.0323; CIV/CS, P3, P5 and P6, empirical *P*-value = 0.0340, 0.0487, 0.0310; citrate synthase, P1, P3, P4, P5 and P6, empirical *P*-value: 0.0318, 0.0360, 0.0261, 0.0348 and 0.0354) (***P*-value ≤ 0.01, **P*-value ≤ 0.05).

© 2020 The Authors
